# Interlayer Spacing Expansion and Active Site Engineering of MXene@TiO_2_ Heterostructures as Robust Cathodes for Rechargeable Aluminum-Ion Batteries

**DOI:** 10.3390/molecules31214014

**Published:** 2026-11-06

**Authors:** Qiongfeng Chen, Wenhui Zhou, Yongshang Zhang, Yongbiao Liu, Linsen Zhang

**Affiliations:** 1 Ruyuan Dongyangguang Fluorine Resin Co., Ltd., Shaoguan 512799, China; 2 College of New Energy, Zhengzhou University of Light Industry, Zhengzhou 450001, China; 3 Henan International Joint Laboratory of Ceramic Energy Materials, Zhengzhou 450001, China; 4 Shanghai Putailai New Energy Technology Co., Ltd., Shanghai 210315, China

**Keywords:** aluminum-ion batteries, Ti_3_C_2_T*_X_* MXene@TiO_2_, heterostructure, hydrothermal modulation, interlayer engineering

## Abstract

Aluminum-ion batteries (AIBs) have become a highly promising energy storage technology due to their high theoretical capacity, cost-effectiveness, and excellent safety. However, there is still a lack of stable and efficient cathode materials capable of reversibly inserting/extracting aluminum complex ions, which constitutes a key challenge in the field. Herein, we propose a facile and controllable hydrothermal strategy to in situ construct Ti_3_C_2_T*_X_* MXene@TiO_2_ heterostructures, and systematically elaborate the regulatory mechanism of hydrothermal temperature and reaction duration on the morphology, interlayer structure, surface chemistry, and electrochemical behaviors of the composites. The results demonstrate that uniformly dispersed anatase TiO_2_ nanoparticles are anchored on MXene nanosheets, which effectively suppresses layer stacking, expands interlayer spacing from 1.040 nm to 1.344 nm, and synchronously regulates surface terminal groups of MXene. Benefiting from the synergistic effect of expanded ion diffusion channels, abundant pseudocapacitive active sites, and robust heterointerfaces, the optimized Ti_3_C_2_T*_x_*@TiO_2_ electrode delivers a high initial reversible capacity of 243.8 mAh g^−1^ and maintains a stable capacity of 80 mAh g^−1^ after 200 consecutive cycles with negligible capacity decay. The proposed structural engineering strategy provides universal guidance for the rational design of high-performance MXene-based electrode materials for next-generation aluminum-ion batteries and other advanced energy storage systems.

## 1. Introduction

The large-scale integration of wind, solar, and hydropower into modern power grids has become an inevitable trend to achieve carbon neutrality and replace fossil fuels [1,2,3]. However, the inherent intermittency and volatility of renewable energy lead to severe grid imbalance, which puts forward stringent requirements for supporting energy storage technologies [4,5,6,7]. Lithium-ion batteries (LIBs) currently dominate the commercial energy storage market, but the scarcity of lithium resources, rising raw material costs, and potential safety hazards severely limit their large-scale deployment in grid-level energy storage. Against this backdrop, rechargeable post-lithium battery systems represented by sodium-ion, magnesium-ion, zinc-ion, and aluminum-ion batteries have attracted worldwide research attention [8,9,10].

Among various emerging battery technologies, aluminum-ion batteries (AIBs) stand out with unparalleled comprehensive advantages [11,12,13]. Aluminum metal features ultrahigh natural abundance, low cost, and excellent operational safety [14,15,16]. More importantly, each Al atom can participate in a three-electron redox reaction, endowing AIBs with an ultrahigh theoretical specific capacity of 2980 mAh g^−1^, far exceeding most monovalent metal-ion battery systems [17,18,19]. Therefore, AIBs are universally recognized as a promising candidate for low-cost, large-capacity, and long-cycle grid energy storage. Nevertheless, the commercialization of AIBs is still plagued by a core bottleneck: the lack of high-performance cathode materials that can realize reversible and stable intercalation/deintercalation of aluminum complex ions (mainly [AlCl_4_]^−^ in chloroaluminate ionic liquid electrolytes).

At present, mainstream AIB cathode materials can be divided into transition metal compounds and carbon-based materials [20,21,22]. Transition metal oxides and sulfides possess relatively high specific capacity, but their rigid crystal structures are prone to irreversible structural destruction under the repeated embedding of large-sized [AlCl_4_]^−^ anions, resulting in rapid capacity fading and poor cycling stability [23,24,25]. Carbon-based layered materials such as graphite and reduced graphene oxide can buffer volume variation via layered structures and suppress side reactions, but their charge storage is mainly dominated by electric double-layer capacitance, leading to low specific capacity and inability to meet high energy density demands [26,27]. The above defects severely restrict the practical application of traditional cathode materials.

Two-dimensional transition metal carbides (MXenes), represented by Ti_3_C_2_T*_x_*, have emerged as a new generation of promising electrode materials for AIBs due to their unique physicochemical properties [28,29]. Metallic conductivity of MXenes ensures rapid electron transmission; hydrophilic surface terminal groups (–OH, –O, –F) guarantee excellent electrolyte wettability and reduce interfacial impedance; tunable layered structure provides intrinsic channels for ion migration [30]. In addition, abundant surface functional groups can offer extra pseudocapacitive active sites to enhance ion storage capacity. A series of studies have verified that MXene materials can achieve stable cycling in AIBs, benefiting from their layered structure and surface functional characteristics [31].

Unfortunately, pristine Ti_3_C_2_T*_x_* MXene still faces three critical drawbacks in AIB application: (1) narrow intrinsic interlayer spacing increases the diffusion barrier of large-size [AlCl_4_]^−^; (2) severe self-stacking of MXene nanosheets reduces the number of exposed active sites; (3) insufficient pseudocapacitive sites lead to unsatisfactory specific capacity [32]. Furthermore, the static diameter of AlCl_4_^−^ (~0.7 nm) is smaller than the pristine MXene interlayer spacing (~0.93 nm), effective intercalation remains hindered by three key factors: (1) abundant surface terminal groups (−F, −O, −OH) drive interlayer restacking via van der Waals forces and hydrogen bonding, sharply reducing the effective accessible channel size; (2) AlCl_4_^−^ carries a solvation shell in the ionic liquid electrolyte, enlarging its kinetic migration radius; (3) the negatively charged MXene surface exerts electrostatic repulsion on anionic AlCl_4_^−^, raising the insertion energy barrier [33].

To address these issues, constructing MXene/metal oxide heterostructures has been proven to be an effective modification strategy. Among numerous metal oxides, TiO_2_ is favored by researchers for its outstanding structural stability, inherent pseudocapacitive characteristics, low cost, and environmental friendliness [34]. Although MXene@TiO_2_ composites have been applied in lithium-ion batteries and supercapacitors, systematic research on hydrothermal synthesis parameter optimization and structure-performance regulation of Ti_3_C_2_T*_x_*@TiO_2_ for AIBs is still insufficient [35,36,37]. The correlation mechanism between hydrothermal conditions (temperature, time), material microstructure, interlayer spacing, surface chemistry, and aluminum ion storage performance has not been clearly elucidated. It remains unclear how to realize precise modulation of TiO_2_ loading state, MXene interlayer structure, and surface functional groups via controllable hydrothermal treatment, which hinders the targeted design of high-performance MXene-based AIB cathodes.

In view of the above research gaps, we herein design and fabricate Ti_3_C_2_T*_x_*@TiO_2_ heterostructured composites via a one-step controllable hydrothermal method, and systematically investigate the effects of hydrothermal temperature (100 °C, 120 °C, 150 °C) and reaction duration (3 h, 6 h, 12 h) on the morphology, crystal structure, interlayer spacing, surface chemical state, and electrochemical performance of the composites. Multiple characterization techniques, including SEM, TEM, XRD, Raman, and XPS, are combined to reveal the structural evolution rule of MXene under different hydrothermal conditions. Electrochemical tests, including cyclic voltammetry (CV), galvanostatic charge–discharge (GCD), rate performance, and electrochemical impedance spectroscopy (EIS), are adopted to clarify the charge storage mechanism and ion transport kinetics of the composites.

The experimental results confirm that the optimal hydrothermal condition (120 °C, 6 h) can realize uniform in situ growth of TiO_2_ nanoparticles on MXene nanosheets, effectively expand MXene interlayer spacing, optimize surface functional groups, and construct robust heterointerfaces. The optimized composite exhibits excellent specific capacity, rate capability, and long-cycle stability as an AIB cathode. This work not only develops a simple, low-cost, and scalable hydrothermal regulation strategy for MXene composite cathodes, but also establishes a clear structure-performance relationship. The research findings provide novel insights and universal design principles for the development of high-efficiency MXene-based electrode materials toward advanced aluminum-ion batteries and large-scale energy storage systems.

## 2. Results

**Figure 1:**
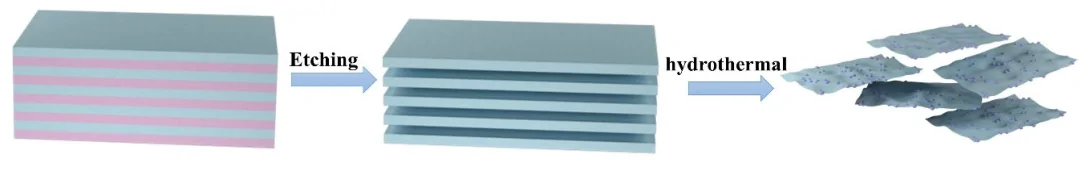
Schematic diagram of the preparation process of Ti_3_C_2_T*_x_*@TiO_2_ heterostructures.

Figure 1 schematically illustrates the entire synthesis procedure of Ti_3_C_2_T*_x_*@TiO_2_ heterostructures, including two core steps: selective etching of the Ti_3_AlC_2_ MAX phase and in situ hydrothermal growth of TiO_2_ nanoparticles on the MXene surface. Firstly, the Al atomic layers in Ti_3_AlC_2_ are completely removed by hydrofluoric acid (HF) etching, obtaining accordion-like layered Ti_3_C_2_T*_x_* MXene with expanded interlayer spacing. Subsequently, TiO_2_ nanoparticles are uniformly grown on the surface and interlayers of MXene via a facile hydrothermal reaction in a formaldehyde-containing solution. By adjusting hydrothermal temperature and reaction time, we realize precise control over the loading amount, dispersion state, and particle size of TiO_2_, as well as the interlayer architecture of MXene.

**Figure 2:**
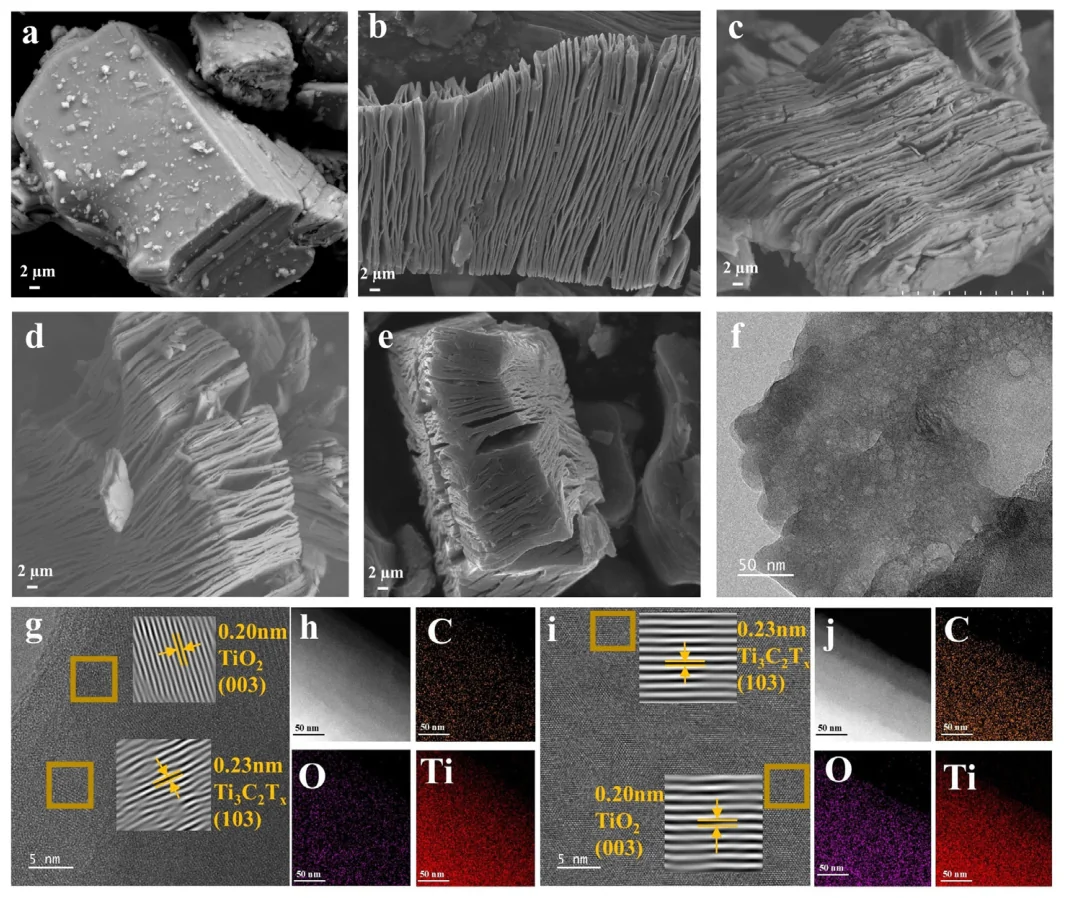
(**a**–**e**) SEM images of Ti_3_AlC_2_ MAX, Ti_3_C_2_ MXene, T120-3h, T120-6h, and T120-12h. (**f**–**j**) TEM images and EDS of T120-6h.

The morphological evolution of samples at different synthesis stages is characterized by scanning electron microscopy (SEM), as shown in Figure 2a–e. The pristine Ti_3_AlC_2_ MAX phase presents a dense bulk layered structure (Figure 2a). After HF selective etching, the interlayer Al atoms are stripped off, and the material transforms into a typical accordion-like multilayer Ti_3_C_2_ MXene (Figure 2b). The expanded interlayer gaps provide sufficient space for the subsequent anchoring of TiO_2_ nanoparticles and migration of [AlCl_4_]^−^ ions.

To screen the optimal hydrothermal temperature, we fixed the reaction time at 12 h and explored the morphological changes in composites at 100 °C, 120 °C, and 150 °C. At 100 °C, the hydrothermal reaction kinetic energy is insufficient, so only a small number of sparse TiO_2_ nanoparticles are sporadically distributed on the MXene surface, which cannot form an effective heterostructure (Figure S1a). When the temperature rises to 120 °C, the reaction rate is moderate, and abundant TiO_2_ nanoparticles are uniformly loaded on MXene nanosheets and interlayers. This uniform coating can effectively inhibit the restacking of MXene layers and expose more active sites (Figure S1b). Upon further increasing the temperature to 150 °C, excessive reaction activity leads to serious agglomeration of TiO_2_ nanoparticles. The large particle clusters will block the ion diffusion channels and reduce the utilization of active materials. Therefore, 120 °C is determined as the optimal hydrothermal temperature.

On the basis of a fixed temperature (120 °C), we further investigate the influence of hydrothermal time (3 h, 6 h, 12 h) on material morphology (Figure 2c–e). For the sample treated for 3 h (T120-3h), the reaction time is too short, TiO_2_ nucleation is incomplete, and nanoparticles are unevenly distributed on the MXene surface (Figure 2c). When the hydrothermal time is extended to 6 h (T120-6h), TiO_2_ nanoparticles grow fully and disperse homogeneously on the MXene surface and interlayers. The formed tight MXene-TiO_2_ heterointerfaces can generate strong synergistic effects, while the intrinsic layered framework of MXene is well preserved (Figure 2d). When the reaction time is prolonged to 12 h (T120-12h), overgrowth of TiO_2_ occurs, accompanied by particle agglomeration and increased particle size in local regions (Figure 2e). The above SEM results demonstrate that hydrothermal time is a key parameter to regulate the dispersion and loading of TiO_2_. The T120-6h sample achieves the optimal morphological structure: uniform TiO_2_ decoration effectively suppresses MXene layer stacking, maintains complete layered structure, and constructs continuous ion/electron transport networks. This structure is highly favorable for the reversible intercalation of large-sized [AlCl_4_]^−^ ions and alleviates volume variation during long-term cycling.

Transmission electron microscopy (TEM) and high-resolution TEM (HRTEM) are used to further analyze the micro-nano structure of the composite. The low-magnification TEM image (Figure 2f) clearly shows the two-dimensional sheet structure of Ti_3_C_2_T*_x_* MXene and TiO_2_ nanoparticles anchored on the surface, confirming the successful combination of the two components. HRTEM images display distinct lattice fringes: the lattice spacing of 0.20 nm corresponds to the (200) crystal plane of anatase TiO_2_, and the lattice fringe of 0.23 nm is assigned to the (103) plane of Ti_3_C_2_T*_x_* MXene (Figure 2g,i). The coexistence of two sets of lattice fringes verifies the formation of intimate heterointerfaces between MXene and TiO_2_. Energy-dispersive X-ray spectroscopy (EDS) elemental mapping reveals the uniform distribution of C, Ti, and O elements across the entire sample, which further proves the homogeneous integration of TiO_2_ and the MXene matrix. Such a well-defined heterostructure can optimize electronic conductivity and ion diffusion kinetics for AIB cathodes. SEM and TEM observations clearly show the morphological evolution: TiO_2_ nanoparticles are sparsely distributed after 3 h of hydrothermal treatment, become uniformly and abundantly loaded at 6 h, and undergo obvious agglomeration and size growth at 12 h. This trend directly reflects the gradual increase in TiO_2_ loading with extended reaction time.

**Figure 3:**
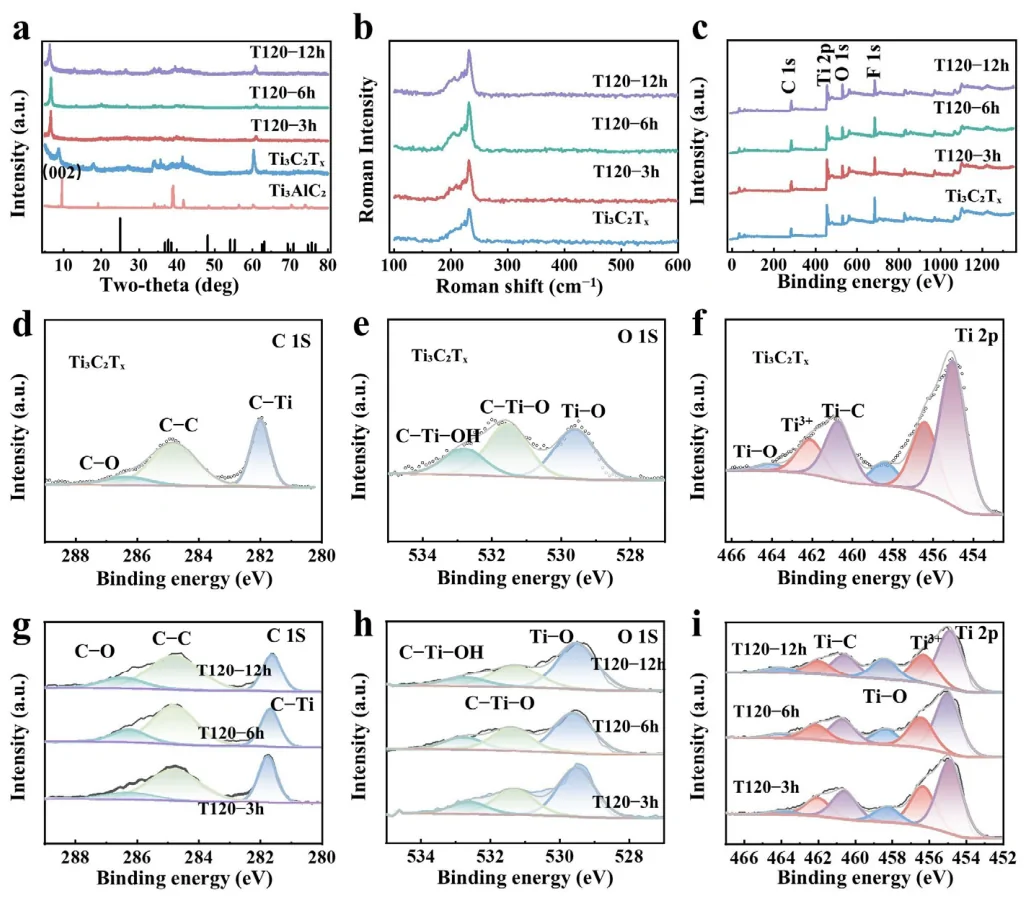
(**a**,**b**) XRD patterns and Raman spectra of Ti_3_C_2_ MXene, T120-3h, T120-6h, and T120-12h. (**c**–**i**) XPS survey spectra of Ti_3_C_2_ MXene, T120-3h, T120-6h, and T120-12h.

X-ray diffraction (XRD) is employed to characterize the crystal phase evolution and interlayer spacing variation in materials during synthesis. As presented in Figure 3a, the pristine Ti_3_AlC_2_ MAX phase shows a series of sharp characteristic diffraction peaks at 2θ = 9.5°, 19.2°, 34.0°, 39.0°, and 41.8°, corresponding to (002), (004), (101), (104), and (105) crystal planes, respectively, which is consistent with standard literature reports. After HF etching, the diffraction peaks related to Al atomic layers near 39.0° completely disappear, indicating the full removal of interlayer Al atoms. Meanwhile, the (002) characteristic peak of MXene shifts from 9.5° to 8.5°. According to Bragg’s law, the interlayer spacing increases from 0.93 nm to 1.040 nm, which directly confirms the formation of expanded layered Ti_3_C_2_T*_x_* MXene.

Low-angle XRD is further used to quantitatively analyze the change in MXene interlayer spacing after different hydrothermal treatments, which is the core structural advantage of our modified materials. For the etched Ti_3_C_2_T*_x_* without hydrothermal treatment, the (002) peak is located at 2θ = 8.50 nm, with an interlayer spacing of 1.040 nm. After hydrothermal treatment at 120 °C for 3 h and 6 h, the (002) diffraction peak continuously shifts toward lower angles. Specifically, the (002) peak of the T120-6h sample moves to 2θ = 6.57°, and the corresponding interlayer spacing expands to 1.344 nm. The significant expansion of interlayer spacing is derived from three synergistic factors: (1) Hydrothermal treatment induces the partial removal of interlayer –F groups and the conversion of surface –OH to –O groups, reducing the steric hindrance between MXene layers; (2) Water molecules are intercalated into MXene interlayers during the hydrothermal process, acting as “pillars” to prop open the layered structure; (3) In situ grown TiO_2_ nanoparticles on the MXene surface introduce steric hindrance, effectively preventing the re-stacking of nanosheets. The expanded interlayer spacing (from 1.040 nm to 1.344 nm) greatly reduces the diffusion resistance of large-size [AlCl_4_]^−^ ions, accelerates ion migration kinetics, and improves the rate performance of the electrode. Notably, excessive hydrothermal time will cause over-oxidation of MXene and excessive growth of TiO_2_, which may destroy the layered structure. The moderate hydrothermal condition (6 h) realizes the controllable expansion of interlayer spacing while maintaining the integrity of the MXene layered framework, which is the key structural foundation for excellent cycling stability.

Raman spectroscopy is used to characterize the molecular vibration and surface structure of Ti_3_C_2_T*_x_* MXene (Figure 3b). The distinct characteristic peak at ~230 cm^−1^ is attributed to the stretching vibration of Ti–C layers and the vibration modes of surface functional groups, which is a typical Raman signal of Ti_3_C_2_T*_x_* MXene. No obvious impurity peaks are observed in the composite samples, proving that the MXene crystal skeleton is well retained after hydrothermal modification.

X-ray photoelectron spectroscopy (XPS) is conducted to explore the surface chemical composition, elemental valence state, and functional group evolution of Ti_3_C_2_T*_x_*@TiO_2_ composites under different hydrothermal durations, revealing the regulatory mechanism of hydrothermal treatment on MXene surface chemistry (Figure 3c–i). Figure 3c shows the XPS survey spectrum of the etched Ti_3_C_2_T*_x_* sample. No characteristic signal of the Al element was observed in the full-range XPS spectrum, indicating that the residual Al content is below the detection limit of the XPS technique. Combined with the complete disappearance of Ti_3_AlC_2_ characteristic diffraction peaks in XRD, these two pieces of evidence mutually support that the Al atomic layers in the MAX phase have been effectively removed after HF etching, with only negligible Al residue retained in the sample. The high-resolution C 1s spectra can be deconvoluted into three characteristic peaks: C–Ti (~282.0 eV), C–C (~284.8 eV), and C–O (~286.3 eV). The C–Ti peak originates from the intrinsic chemical bond of the MXene skeleton; C–C is derived from surface-adsorbed carbon species; C–O corresponds to oxygen-containing functional groups on the MXene surface (Figure 3d,g). With the extension of hydrothermal time, the peak area of C–Ti decreases gradually, while the area of C–C increases, indicating that hydrothermal treatment induces partial surface oxidation and functional group transformation of MXene. The O 1s spectra are fitted into three components: Ti–O (~529.6 eV), C–Ti–O (~531.4 eV), and C–Ti–OH (~532.7 eV). Ti–O represents lattice oxygen of anatase TiO_2_; C–Ti–O corresponds to the interfacial oxygen between MXene and TiO_2_; C–Ti–OH is assigned to surface hydroxyl groups of MXene (Figure 3e,h). As hydrothermal time increases, the peak area of Ti–O rises continuously, which is consistent with the gradual increase in TiO_2_ content. Meanwhile, the signal of C–Ti–OH weakens, demonstrating that surface hydroxyl groups are gradually substituted and transformed during the hydrothermal reaction.

The Ti 2p spectra (Figure 3f,i) are divided into three valence states: Ti–C (MXene matrix), Ti^3+^ (surface defect state), and Ti–O (TiO_2_). The peak area of Ti–C and Ti^3+^ decreases with prolonged hydrothermal time, while the Ti–O signal is significantly enhanced, proving the gradual oxidation of Ti species and in situ generation of TiO_2_. For the T120-6h sample, the attenuation of Ti–C and Ti^3+^ signals is moderate, and the growth of the Ti–O signal is at a medium level. This result indicates that moderate hydrothermal treatment forms a thin and uniform TiO_2_ coating on the MXene surface, rather than an excessively thick oxide layer. This mild surface oxidation not only introduces abundant pseudocapacitive active sites from TiO_2_, but also fully preserves the high conductivity and structural integrity of the MXene skeleton. In contrast, the 12 h hydrothermal sample suffers from over-oxidation, which damages the MXene conductive network. In summary, the 6 h hydrothermal treatment achieves an optimal balance between MXene surface oxidation degree and structural stability, realizing the dual regulation of interlayer structure and surface functional groups. This is another core innovation of this work for optimizing AIB cathode performance.

**Figure 4:**
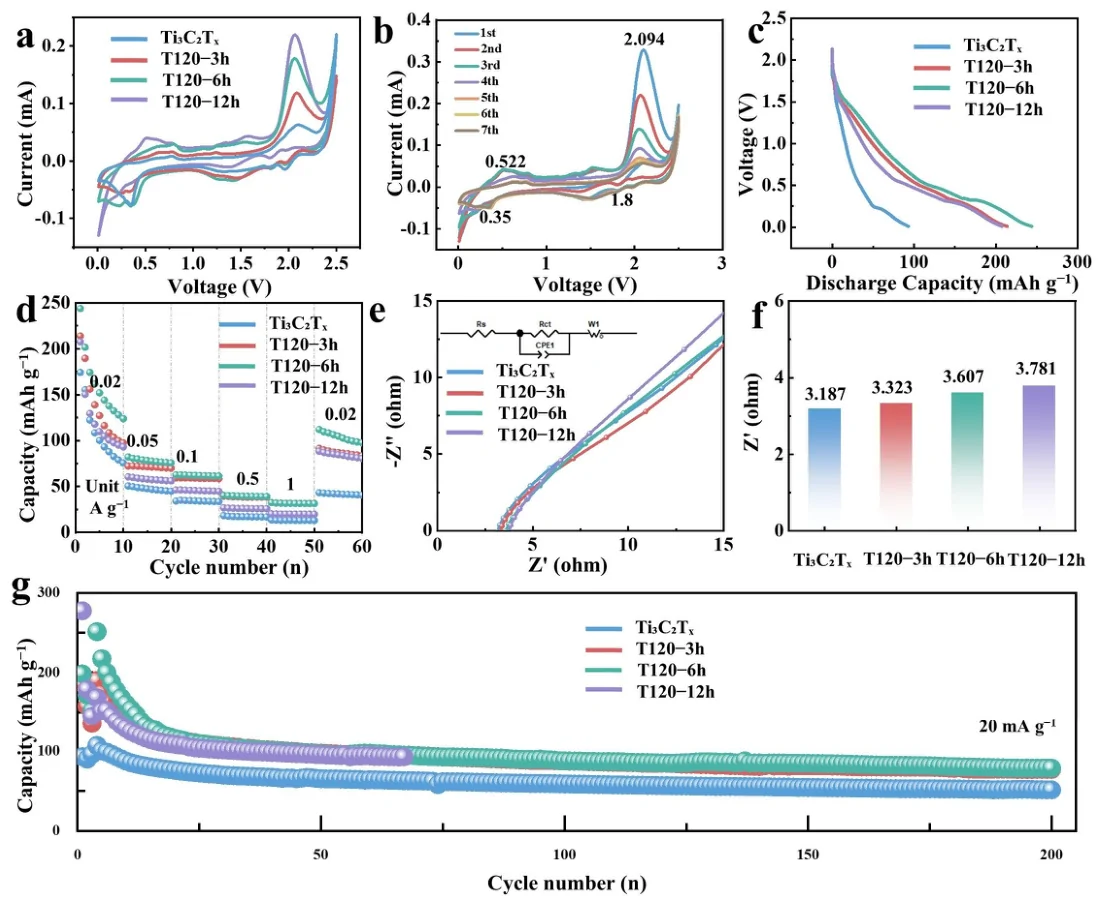
(**a**,**b**) CV curves of Ti_3_C_2_ MXene, T120-3h, T120-6h, and T120-12h. (**e**,**f**) Nyquist EIS plots of Ti_3_C_2_ MXene, T120-3h, T120-6h, and T120-12h. (**c**,**d**,**g**) Galvanostatic charge–discharge profiles, rate performance, and cycling performance of Ti_3_C_2_ MXene, T120-3h, T120-6h, and T120-12h.

Cyclic voltammetry (CV) tests at a scan rate of 0.1 mV s^−1^ are performed to investigate the electrochemical reaction behavior and reversibility of electrodes (Figure 4a and Figure S2). The CV curves of all samples present the combined characteristics of rectangular shape and redox peaks, indicating that the charge storage of Ti_3_C_2_T*_x_*@TiO_2_ composites follows the synergistic mechanism of electric double-layer capacitance and pseudocapacitance/intercalation-type battery behavior. For the samples treated at 120 °C with different durations, the CV integral area and redox peak current follow the order: T120-6h > T120-3h > T120-12h > pristine Ti_3_C_2_T*_x_*. The T120-6h sample possesses the largest CV area and the strongest redox peak response, which manifests the highest electrochemical activity, optimal [AlCl_4_]^−^ intercalation/deintercalation kinetics, and charge transfer capability. The short-time hydrothermal (3 h) cannot form sufficient active sites, while over-treatment (12 h) leads to structural damage and ion channel blockage, resulting in decreased electrochemical activity.

Multiple consecutive CV cycles of T120-6h are tested to evaluate cycling reversibility (Figure 4b). The CV curves from the 2nd to 7th cycle overlap almost completely, with stable redox peak positions and current intensity. Two oxidation peaks at 0.52 V and 2.1 V, and two reduction peaks at 0.35 V and 1.8 V are observed, corresponding to the reversible embedding and extraction of [AlCl_4_]^−^ ions. The slight difference between the first cycle and subsequent cycles is attributed to the irreversible electrolyte wetting and initial functional group transformation. The highly overlapping CV curves strongly prove the excellent electrochemical reversibility and structural stability of the T120-6h electrode.

Galvanostatic charge–discharge (GCD) tests are carried out at a current density of 20 mA g^−1^. The first-cycle discharge specific capacities of samples hydrothermally treated at different temperatures (100, 120, 150 °C) for 12 h are shown in Figure S3. It can be seen that T120-12h exhibits the highest discharge specific capacity. For this temperature (120 °C), different hydrothermal times were further investigated, and the corresponding voltage-discharge capacity curves are presented in Figure 4c. The pristine Ti_3_C_2_T*_x_* only delivers a low initial discharge capacity of 93.1 mAh g^−1^. After hydrothermal modification, the specific capacity is greatly improved: T120-3h (213.9 mAh g^−1^), T120-6h (243.8 mAh g^−1^), and T120-12h (207.6 mAh g^−1^). The T120-6h sample exhibits the highest initial capacity, which is consistent with CV results, further verifying that moderate hydrothermal regulation maximizes the number of active sites and optimizes ion transport pathways. The cyclic voltammetry curves exhibit a combined feature of a quasi-rectangular profile (characteristic of electric double-layer capacitance and surface pseudocapacitance) and distinct redox peaks (corresponding to Faradaic intercalation of [AlCl_4_]^−^), which is a typical signature of coexisting capacitive and diffusion-controlled behaviors. The galvanostatic charge–discharge profiles show sloped voltage curves without long flat plateaus, further confirming that charge storage arises from both surface capacitive processes and bulk ion intercalation, rather than a single mechanism. The rate performance trend also supports this inference: at low current densities, the electrode delivers higher specific capacity dominated by diffusion-limited intercalation behavior; as current density increases, the capacitive process gradually dominates, enabling relatively stable cycling and rate capability.

Long-term cycling performance is a key index for AIB cathode practical application. As shown in Figure 4g, at 20 mA g^−1^, the pristine Ti_3_C_2_T*_x_* electrode undergoes rapid capacity decay, and the capacity drops to only 51 mAh g^−1^ after 200 cycles, caused by severe layer stacking and structural degradation during cycling. By contrast, the T120-6h electrode retains a reversible capacity of 80 mAh g^−1^ after 200 cycles, with a flat cycling platform and capacity retention of ~32.8%. In our composite, TiO_2_ nanoparticles are tightly anchored on the MXene surface via in situ hydrothermal growth. From the ex situ SEM and XPS data of the fresh electrode, TiO_2_ is uniformly distributed on MXene nanosheets. During repeated charge/discharge cycles, two competing trends exist: (1) The rigid TiO_2_ nanoparticles can act as physical pillars to stabilize the MXene layered structure and mitigate large-scale restacking; (2) Long-term ion intercalation/deintercalation and volume fluctuation may induce partial detachment or local aggregation of TiO_2_ nanoparticles on the MXene surface, which is one of the main reasons for capacity decay.

The excellent long-cycle stability benefits from three factors: (1) Expanded interlayer spacing buffers the volume change during ion intercalation; (2) uniform TiO_2_ coating suppresses MXene structural collapse; (3) robust heterointerfaces maintain stable electrochemical reaction interfaces. Rate performance tests show that the T120-6h electrode delivers higher reversible capacities at all current densities (20, 50, 100, 500, 1000 mA g^−1^) compared with pristine MXene. When the current density returns to 20 mA g^−1^, the capacity can recover well, demonstrating outstanding rate adaptability and structural robustness (Figure 4d and Figure S4). Furthermore, we summarize the key electrochemical metrics (specific capacity, cycling stability, and rate performance) of previously reported cathode materials for aluminum-ion batteries and compare them with our Ti_3_C_2_T*_x_*@TiO_2_ composite, as shown in Table S1. The comparison demonstrates that our hydrothermally optimized Ti_3_C_2_T*_x_*@TiO_2_ composite delivers competitive electrochemical performance. The introduced TiO_2_ interlayer spacer improves the utilization of MXene active sites and facilitates AlCl_4_^−^ transport.

Electrochemical impedance spectroscopy (EIS) is used to analyze the interfacial reaction kinetics. The intercept of the Nyquist plot on the real axis represents the equivalent series resistance (*R_s_*). All samples have similar *R_s_* values, indicating that hydrothermal treatment has little effect on electrolyte wettability and bulk conductivity of electrodes (Figure 4e,f and Figure S5). The semicircle in the high-to-medium frequency region corresponds to charge transfer resistance (*Rct*). The T120-6h sample presents the smallest *Rct*, proving that the optimized heterostructure significantly accelerates interfacial charge transfer and ion diffusion.

Combined with all electrochemical results, we conclude that the charge storage mechanism of the Ti_3_C_2_T*_x_*@TiO_2_ composite is dual-mode: battery-type ion intercalation dominates at low current density to realize high specific capacity; pseudocapacitive behavior plays a leading role at high current density to guarantee excellent rate capability and cycling stability. This dual-mode mechanism endows the composite with comprehensive electrochemical performance superior to single-component materials.

## 3. Materials and Methods

**Preparation of MXenes.** Firstly, 3 g of Ti_3_AlC_2_ MAX phase powder was gradually and repeatedly added to 30 mL of 48% HF solution. The mixture was then placed in a hydrothermal reactor and subjected to an etching process at 35 °C for 24 h. After etching, the resulting mixture was washed with deionized water by repeated centrifugation (5000 rpm, 5 min) to remove residual HF and impurities until the pH of the supernatant reached approximately 6, yielding Ti_3_C_2_T*_x_* MXene bulk material with an accordion-like structure.

**Preparation of T100/T120/T150-12h.** At first, 0.4 g of titanium carbide was weighed and mixed with 0.6 mL of deionized water and 2.5 mL of DMAC, followed by ultrasonication for 20 min. Then, 1.42 g of formaldehyde was added under stirring, and the mixture was ultrasonicated for another 10 min. The mixture was stirred in a water bath at 80 °C for 1 h. After cooling to room temperature under stirring, 2.3 mL of hydrochloric acid was added to adjust the pH to below 3. Subsequently, hydrothermal treatment was carried out at 100 °C, 120 °C, and 150 °C for 12 h, respectively. After cooling, the product was rinsed with anhydrous ethanol, ultrasonicated for 20 min, then washed with anhydrous ethanol and filtered under suction (twice). The obtained sample was dried under vacuum at 60 °C for 6 h.

**Preparation of T120-3h/6h/12h.** First, 0.4 g of titanium carbide was weighed and mixed with 0.6 mL of deionized water and 2.5 mL of DMAC, followed by ultrasonication for 20 min. Then, 1.42 g of formaldehyde was added under stirring, and the mixture was ultrasonicated for another 10 min. The mixture was stirred in a water bath at 80 °C for 1 h. After cooling to room temperature under stirring, 2.3 mL of hydrochloric acid was added to adjust the pH to below 3. Subsequently, hydrothermal treatment was carried out at 120 °C for 3 h, 6 h, and 12 h, respectively. After cooling, the product was rinsed with anhydrous ethanol, ultrasonicated for 20 min, then washed with anhydrous ethanol and filtered under suction (twice). The obtained sample was dried under vacuum at 60 °C for 6 h.

**Assembly and electrochemical measurement of aluminum-ion batteries.** The preparation of the cathode material for AIBs involved mixing Ti_3_C_2_T_x_ MXene, carbon black powder, and polyvinylidene fluoride (PVDF) binder at a mass ratio of 8:1:1 using N-methyl-2-pyrrolidone (NMP, 99%, Alfa Aesar, Haverhill, MA, USA) as the solvent. All electrodes used Ti_3_C_2_T_x_@TiO_2_ composites as active materials, and the mass ratio of active composite: carbon black: PVDF was fixed at 8:1:1 for every sample in this study. The resulting slurry was then directly drop-cast onto a molybdenum foil current collector to form the cathode material, ensuring an active material loading of 1.0–1.5 mg cm^−2^ per cell. The chloroaluminate ionic liquid electrolyte was prepared by mixing 1-ethyl-3-methylimidazolium chloride ([EMIm]Cl, 99.9%) and anhydrous high-purity aluminum chloride (AlCl_3_, 99.99%) at a molar ratio of 1:1.3. Finally, the MXene cathode, aluminum (Al) anode, AlCl_3_/[EMIm]Cl electrolyte, and a Whatman GF/C microporous membrane separator were assembled to complete a Swagelok-type Al battery in a glovebox under an inert atmosphere.

## 4. Conclusions

In this work, we successfully fabricated Ti_3_C_2_T*_x_*@TiO_2_ heterostructured composites via a controllable one-step hydrothermal method, and systematically revealed the regulatory mechanism of hydrothermal temperature and reaction time on the morphology, crystal structure, interlayer spacing, surface chemistry, and electrochemical performance of the composites for aluminum-ion battery cathodes. A series of advanced characterization techniques and electrochemical tests confirm that the hydrothermal condition of 120 °C for 6 h is the optimal parameter.

This study clarifies the intrinsic correlation between hydrothermal synthesis parameters, material microstructure, and aluminum ion storage performance. The proposed hydrothermal dual-regulation strategy of interlayer structure and surface chemistry provides a simple, low-cost, and scalable route for the modification of MXene-based electrode materials. More importantly, the structure-performance mechanism summarized in this work offers a valuable reference for the rational design of high-performance MXene composites applied in aluminum-ion batteries and other post-lithium energy storage devices. In future research, we will further optimize the composite system and combine heteroatom doping to continuously improve the energy density and cycling life of AIBs, promoting their practical application in large-scale energy storage.

## Data Availability

The data presented in this study are available on request from the corresponding author. The data used to support the findings of this study can be made available by the corresponding author upon request.

## Supplementary Materials

The following supporting information can be downloaded at: https://www.mdpi.com/article/10.3390/molecules31214014/s1, Figure S1: (a) SEM image of T100-12h MXene. (b) SEM image of T120-12h MXene; Figure S2: CV curves of Ti_3_C_2_ MXene at different temperatures; Figure S3: First discharge specific capacity of Ti_3_C_2_ MXene at different temperatures; Figure S4; Rate performance of Ti_3_C_2_ MXene at different temperatures; Figure S5: Nyquist EIS plots and of Ti3C2 MXene at different temperatures; Table S1: The electrochemical comparison with recent publications [36,37].
