# Supplementary material for: Interlayer Spacing Expansion and Active Site Engineering of MXene@TiO2 Heterostructures as Robust Cathodes for Rechargeable Aluminum-Ion Batteries
Source: Molecules. 2026 Nov 6;31(21):4014. doi: 10.3390/molecules31214014 (PMC13764877; doi:10.3390/molecules31214014)
Supplement: Supplementary file 1 [file molecules-31-04014-s001.zip › molecules-4626831-supplementary.pdf]

Supporting Information

Interlayer Spacing Expansion and Active Sites Engineering of MXene@TiO_2 Heterostructures as Robust Cathodes for Rechargeable Aluminum-Ion Batteries

Qiongfeng Chen [1], Wenhui Zhou [2,3], Yongshang Zhang [2,3,*], Yongbiao Liu [4,*], and Linsen Zhang [2,3,*]

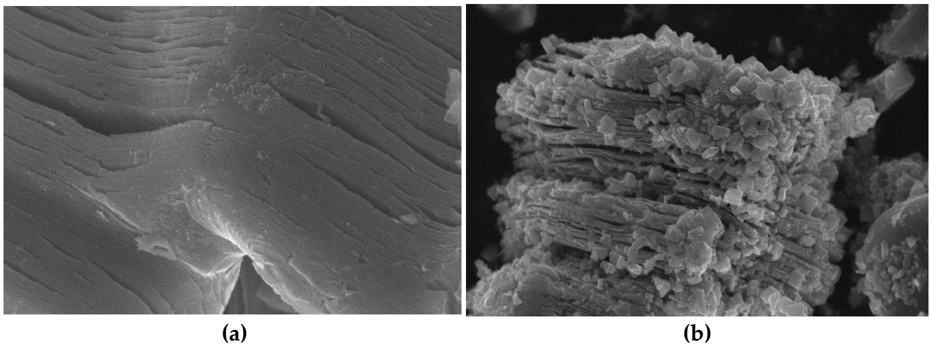

Figure S1. (**a**) SEM image of T100-12h MXene. (**b**) SEM image of T120-12h MXene.

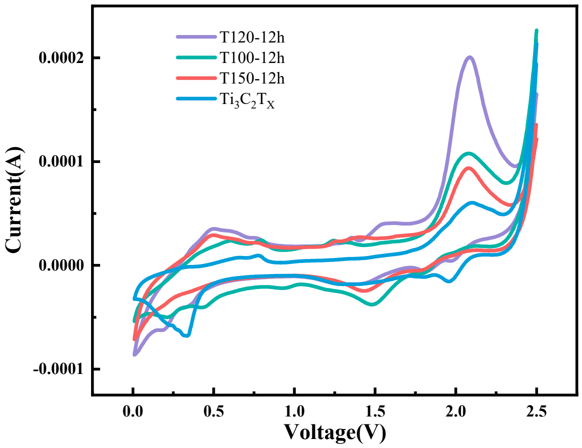

Figure S2. CV curves of Ti_3C_2 MXene at different temperatures.

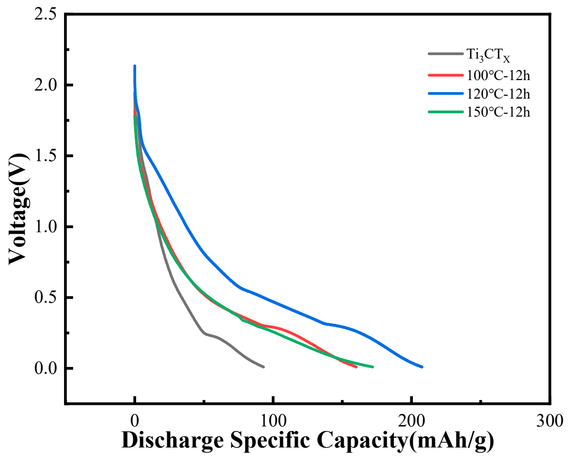

Figure S3. First discharge specific capacity of Ti_3C_2 MXene at different temperatures.

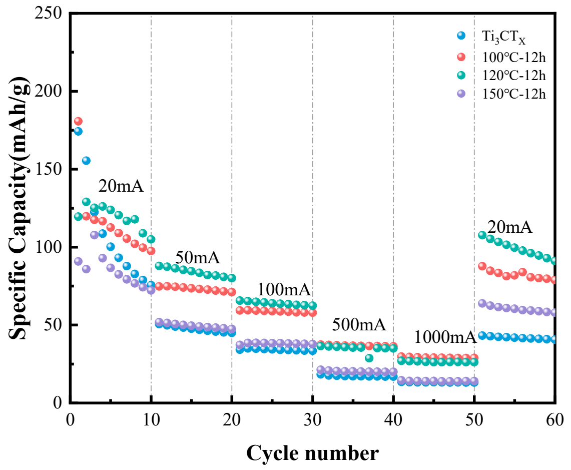

Figure S4. Rate performance of Ti_3C_2 MXene at different temperatures.

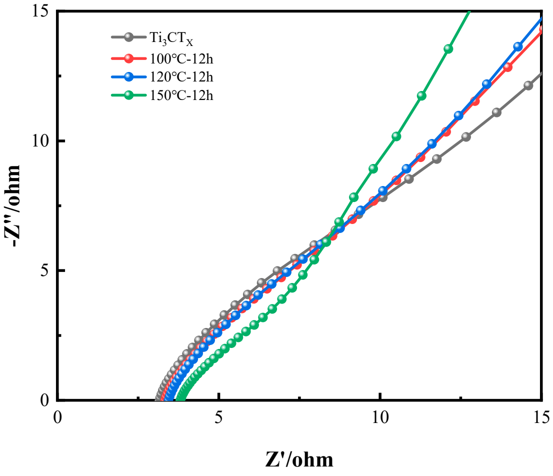

Figure S5. Nyquist EIS plots and of Ti_3C_2 MXene at different temperatures.

Table S1. The electrochemical comparison with recent publications.

Electrolyte composition	Initial Capacity (mAh g^{-1})	Rate Performance (mAh g^{-1})	Cycles	Reversible Capacity (mAh g^{-1})	Ref.
Al(OTF)3/ TU/ TEOS	74.9	56.1 at 0.15 A g^{-1}	100	67.4	1
$AlCl_3$/Et_3NHCl Eetyltrimethyl-ammonium chloride (CTAC)	92	70 at 2 A g^{-1}	600	74.7	2
AlCl3/Dipropyl ether	55	55 at 0.2 A g^{-1}	300	42	3
$AlCl_3$/EMImCl Fluorobenzene (FB)	55	50 at 0.1 A g^{-}	50	60	4
$AlCl_3$/EMImCl	103	85 at 0.1 A g^{-1}	100	85	5
**$AlCl_3$/EMImCl**	**243.8**	**59.5 at 0.5 A g^{-1}**	**200**	**80**	**This work**

References

[1] Z. Wen, F. Wu, M.-F. Ng, B. Jia, J. Song, T. Yu, J. Dong, A. Tang, R. Chen, Q. Yan, Lean-Water Hydrogel with Multipolar Sites for Flexible and High-Performance Aqueous Aluminum Ion Batteries. Adv. Mater. 2025, 37, 2500695.

[2] Y. Xie, X. Du, Y. Meng, Y. Liu, S. Wang, W. You, M. Liu, Y. Guo, Z. Liang, D. Li, Dynamic molecular adsorption interface strategy for stable aluminum batteries, Energy Storage Mater. 2024, 70, 103545.

[3] B. Zhang, Z. Li, D. Han, Z. Min, C. Cui, L. Wang, Q. Li, X. Mao, Y. Sun, Z. Weng, Q.-H. Yang, Non-corrosive organodichloro electrolyte for reversible aluminium metal batteries, Nature Sus. 2026, 9, 295–305.

[4] J. Guo, S. Gu, W. Nie, B. Long, S. Ryazantsev, S. Malyshev, J. Li, S. Guo, C. Wu, Mixed Electrolyte Enabling Ultrafast Mass Transport and Compatibility with Polypropylene Separator for Stable and Low-Cost Aluminum Ion Battery, Adv. Mater. 2025, 37, 2419865.

[5] B. Long, F. Wu, Y. Li, H. Yang, W. Liu, Y. Li, Q. Li, X. Feng, Y. Bai, C. Wu, Manipulating the corrosion homogeneity of aluminum anode toward long-life rechargeable aluminum battery, Carbon Neutral. 2024, 3, 64–73.
